# Femoral head disengagement from Accolade femoral stem in MOM Arthroplasty: a case study and literature review

**DOI:** 10.1051/sicotj/2019030

**Published:** 2019-08-20

**Authors:** Mohamed F. H. Elsheikh, Rehan Gul

**Affiliations:** 1 Department of Trauma and Orthopaedic, Cork University Hospital Wilton, Cork Republic of Ireland

**Keywords:** Metal on Metal Arthroplasty, Trunnionosis, Pseudotumor

## Abstract

*Background*: Metal on Metal Hip arthroplasty was commonly used until the last decade. However Hip Arthroplasty registries of many countries showed increased revision rates of MOM Hips – these high rates of revision caused by trunnionosis, adverse tissue reactions (ALTRs) and Pseudotumor formation.

*The Case*: Our Case is 73 years old gentleman who had left MOM THR in 2008, and was doing fine till the beginning of 2018 when his left leg stuck while getting out of the car. Despite he heard the pop and his leg was shortened and externally rotated, but he was still walking on it for a while. On reviewing him in our orthopedic clinic and after getting the CT-scan showed that he had dislocated femoral head from the stem.

## Introduction

Metal-on-metal (MOM) total hip arthroplasty using cobalt-chrome alloy has been in use since the 1960s as a stemmed replacement [1]. It was popularized due to its enhanced wear profile and the ability to use large femoral heads to reduce post-operative instability [2].

The volumetric wear of MOM is extremely lower than metal on ceramic or polyethylene. However, the particles generated by MOM bearing are significantly smaller than particles of Metal on poly [3, 4].

Metal on Metal Arthroplasty was used commonly until 15 years ago. However, National Registry Data in Australia [5], England, Wales, and Northern Ireland [6], showed that Metal on Metal Hip replacements and resurfacings have significantly higher revision rates compared to Metal on Polyethylene Hip replacements.

The increased rate of MOM arthroplasty revision is thought to be due to the adverse reaction of metal debris released from bearing surface with the surrounding soft tissues which lead to the formation of “pseudotumor” [2].

Trunionosis is another acknowledged cause of Metal on Metal hip arthroplasty failure [7], defined as wear of femoral head–neck interface. It is estimated to account for around 3% of all revision Total hip arthroplasty [8].

The exact causes of trunnionosis are still poorly understood. However, it is believed that the main factors contributing to its occurrence include wear and corrosion at the modular neck–stem taper junction [9–11]. Additionally, Banerjee et al. [12] found that different implant designs and geometries have demonstrated a predisposition to trunnion failure.

Corrosion debris produced at the trunnion Leads to local soft tissue reactions [13, 14], these reactions are clinically and histologically similar to the adverse tissue reactions (ALTRs) seen in MOM [15, 16] and non-MOM bearings [16, 17]. Davies et al. [18] did a comparative study of periprosthetic tissues which obtained at the time of revision of twenty-five cobalt chromium-on-cobalt chromium, nine cobalt chromium-on-polyethylene, and ten titanium-on-polyethylene total hip arthroplasties. Control tissues were obtained from nine osteoarthritic hips at the time of primary total hip arthroplasty, the results showed 68% of the MOM tissues showed perivascular infiltration, and accumulation of plasma cells in association with macrophages that contained metallic wear-debris particles in 40%.

Pseudotumor is another well known MOM-Arthroplasty adverse reaction, it is defined as non-neoplastic and non-infectious cystic or solid mass associated with a hip arthroplasty [19], the exact natural course of its occurrence is still poorly understood [20]. Patients complain most often of groin pain, hip discomfort, paresthesia, antalgic gait, and/or a palpable mass [21, 22]. However, the actual rate of THA revision due to symptomatic pseudotumor is only 1.7–5.6% [21, 23].

## The case

Our patient was a 73-year male, and had Mitch Accolade (MoM) total hip replacement (Product of Stryker Company, HQ Based in Michigan, USA) in 2008; he was doing well with no issues or complains until early Jan 2018 when his left leg unfortunately became stuck under the steering wheel while getting out of car; he twisted his left leg and felt a “pop”.

Since then he was complaining of crepitations in his left hip as well as shortening in his left leg length, yet he was mobilizing on it.

**Figure 1:**
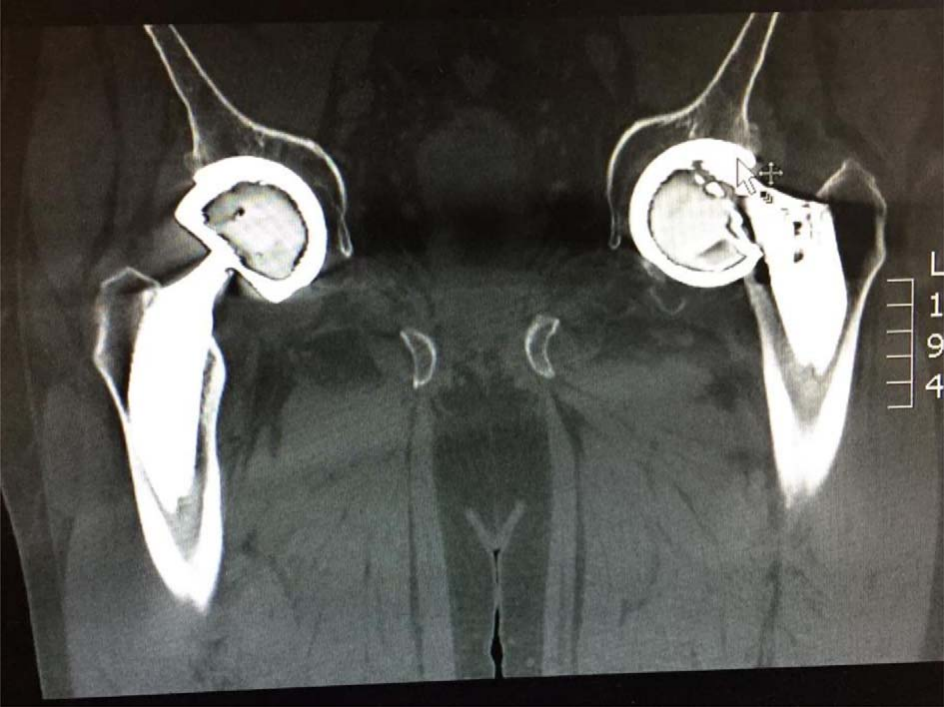
CT scan (Coronal View) showing dislocated femoral head for the trunnion of the femoral neck.

**Figure 2:**
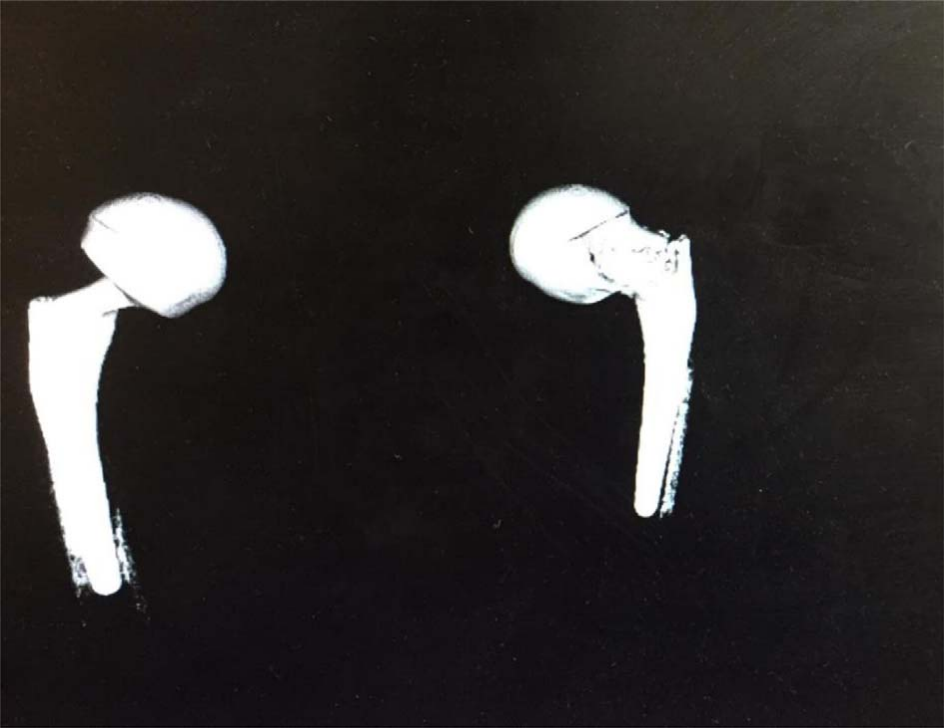
CT scan (3D Construction), showing the dislocation.

He had been reviewed at the orthopedic clinic afterward, where a CT scan was performed (Figures 1 and 2) which confirmed implant dissociation.

His Chromium ion level was 70.2 nmol/L – 3.65 ppb (elevated above 135 nmol/L – 7 parts per billion) and his Cobalt level was 253.45 nmol/L – 14.93 ppb (elevated above 120 nmol), which was elevated.

**Figure 3:**
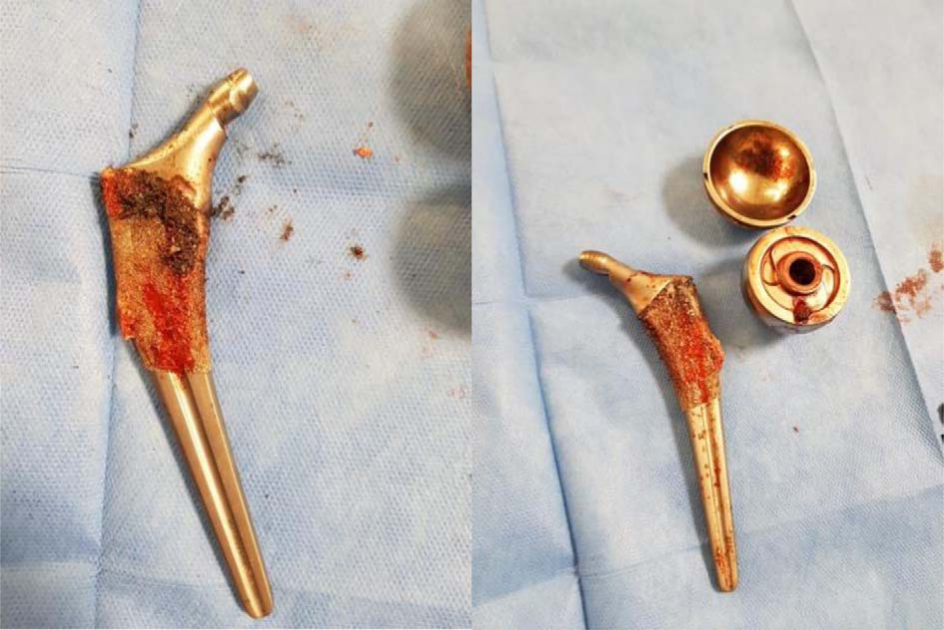
Intra-op pictures of the prothesis, showing the bend at the neck-taper junction.

The patient underwent revision left total hip replacement. Intra-op findings included: a large pseudotumor around the prothesis containing metal debris which was excised, advanced corrosion around the trunnion with deformed shape (Bending at the neck-taper junction, Figure 3) and disengagement of the femoral head from the femoral stem.

**Figure 4:**
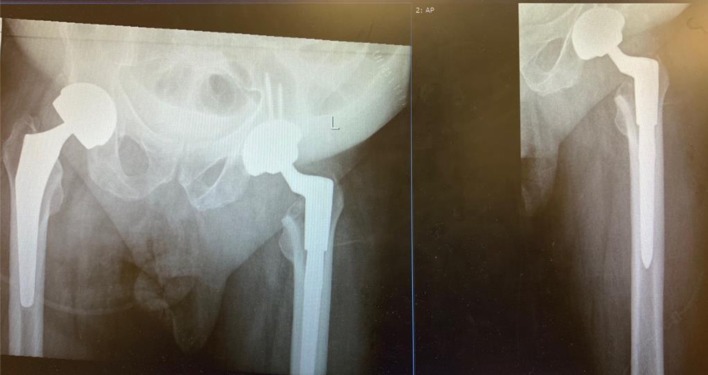
AP pelvis and left hip post-operative check X-rays.

Reclaim conical cementless femoral stem at revision surgery with a pinnacle (Gripton) acetabular shell (Product of DePuy Synthes Company – HQ based in Raynham, USA) was used (Figure 4).

Post-op, the patient did very well with physio and rehab, and he was discharged home a few days later.

## Conclusion

MOM Bearings on Hip Arthroplasty were popular and were commonly used world-wide due to its enhanced wear profile and post-op stability. However, this enthusiasm has declined significantly recently because of its serious complications, such as trunnionosis, elevated serum metal ion levels, aseptic lymphocyte-dominated vasculitis-associated lesions (ALVAL), pseudotumor formation, and subsequent soft tissue and bone destruction.

Trunnionosis is a well-known complication of MOM Total Hip Arthroplasty, the exact cause of which is still poorly understood. However, it is believed that contributing factors include wear between MOM modular junctions [1], corrosion and fretting damage [2], and the release of metal ions or particulate debris from affected components [3].

Failure of the bi-modular stems with cobalt-chrome necks and titanium taper has been widely reported [24–28]. The mechanism of failure of the bi-modular stems is mainly MACC (Mechanically assisted Cervice Corrosion) between the taper and the neck.

To our knowledge, in all prior reported cases of bi-modular femoral stem failures, patients have had broken the femoral stem at neck–taper junction with high serum ion levels of cobalt and chromium. Here is another example of Bi-modular stem failure that presented as head disengagement from the stem without a fracture at the taper–neck junction. We believe this is the only example of Metal on metal modular stem failure that involves head disengagement from the stem without fracture of the taper–neck junction.

## Conflict of interest

The authors declare that they have no conflict of interest.

## Financial disclosure

The authors declare that they have no financial disclosure.
